# Supplementary material for: The Effect of Elevation Gradient on Distribution and Body Size of Carabid Beetles in the Changbaishan Nature Reserve in Northeast Asia
Source: Insects. 2024 Sep 11;15(9):688. doi: 10.3390/insects15090688 (PMC11432507; doi:10.3390/insects15090688)
Supplement: Supplementary file 1 [file insects-15-00688-s001.zip › insects-3175005-supplementary Figure S1.pdf]

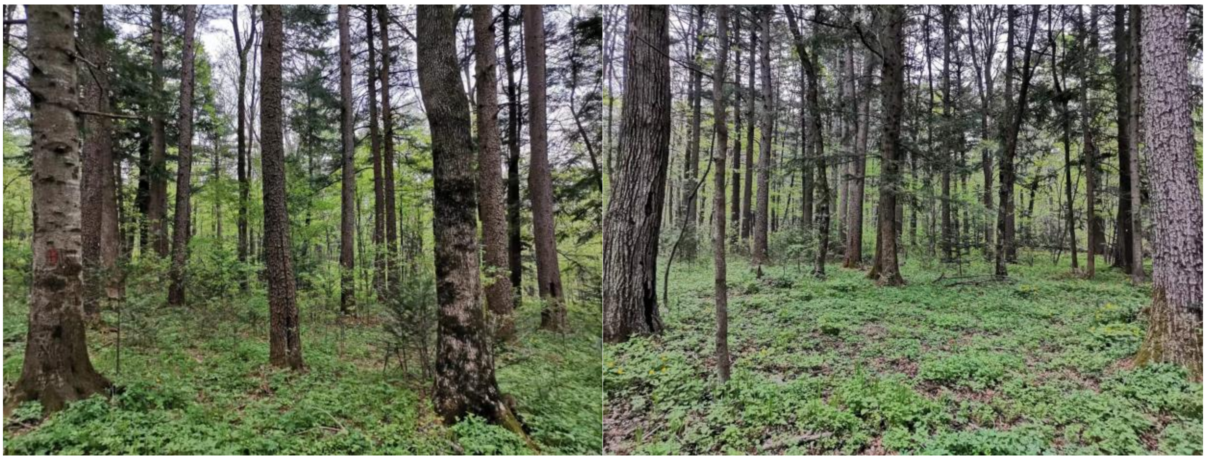

A: Broad leaved Korean pine forest (750–1100 m asl)

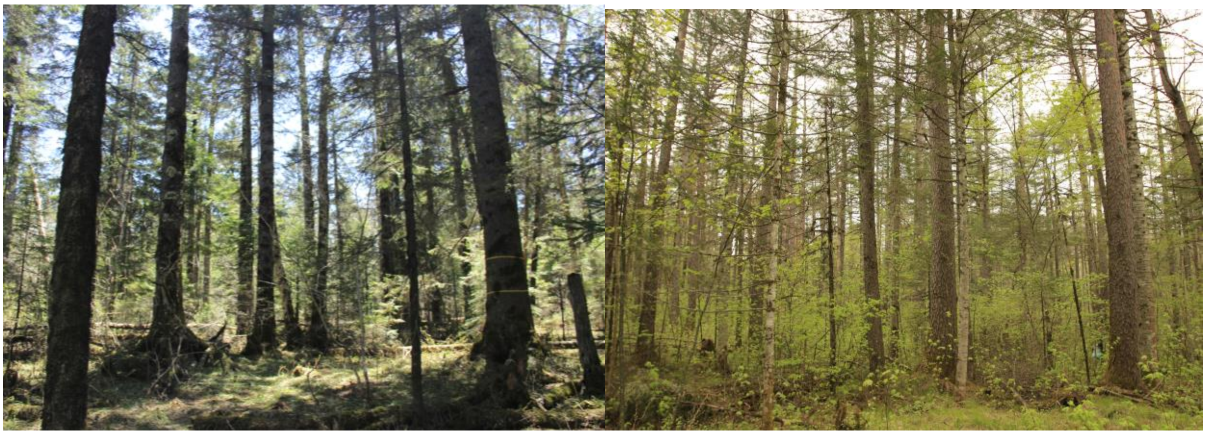

B: Spruce-fir forest (1100–1800 m asl)

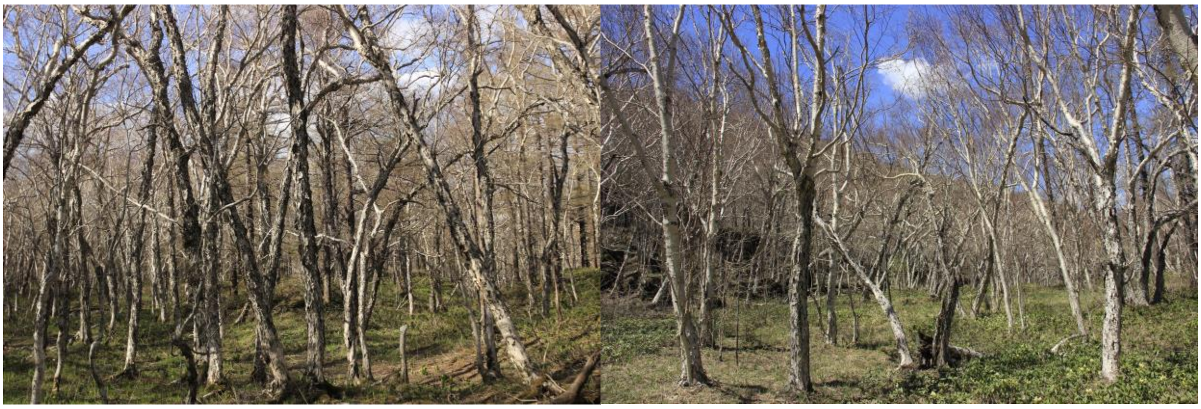

C: Subalpine birch forest (1800–2000 m asl)

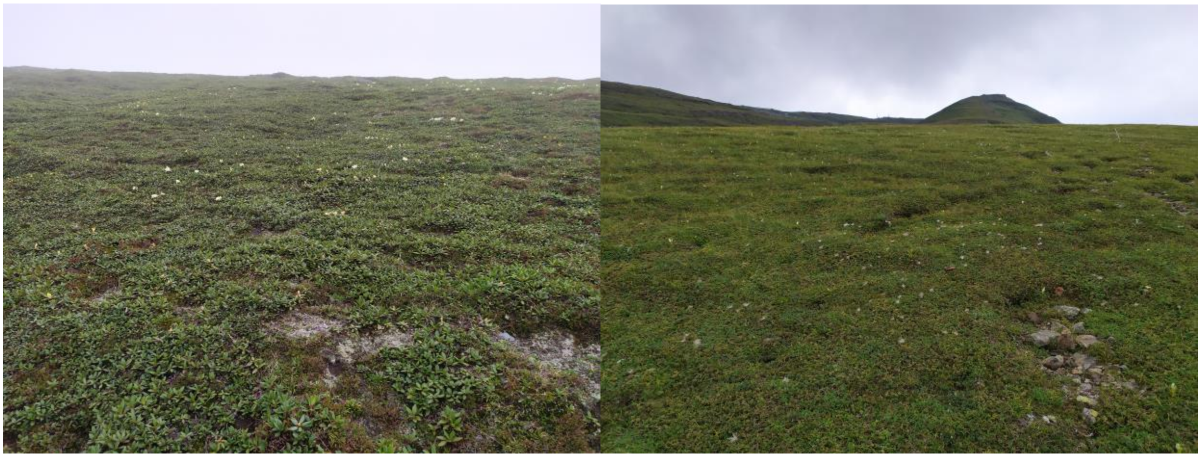

D: Alpine tundra (2000–2600 m asl)

Figure S1 4 vegetation types in Changbaishan Nature Reserve.
