# Supplementary material for: The Effect of Elevation Gradient on Distribution and Body Size of Carabid Beetles in the Changbaishan Nature Reserve in Northeast Asia
Source: Insects. 2024 Sep 11;15(9):688. doi: 10.3390/insects15090688 (PMC11432507; doi:10.3390/insects15090688)
Supplement: Supplementary file 1 [file insects-15-00688-s001.zip › insects-3175005-supplementary Table S1-Table S3.pdf]

Table S1 Transects information of 11 elevations in forest. The value is the mean and standard deviation of the 4 sample plots at each elevation.

Sample abbreviation	Elevation （m）	Area at DBH (m^2)	Average DBH(cm)	Canopy density	Average tree high (m)	Thickness of litter (mm)	Trees number	Tree species composition (Ratio of area at DBH)
A1	750	2.64±0.81	28.44±5.63	0.84±0.03	24.14±0.74	23.5±1.29	22.25±6.7	*P. koraiensis*: *T. amurensis*: *F. mandshurica*: *A. mono*: Other = 3:3:2:1:1
A2	890	1.61±0.48	19.29±3.52	0.82±0.02	22±0.82	23.5±4.36	35.75±4.5	*P. koraiensis*: *P. jezoensis* var. *komarovii*: *A. nephrolepis*: *Q. mongolica*: *T. amurensis*: Other = 2:1:1:3:2:1
A3	1050	2.23±0.63	19.28±2.26	0.84±0.02	22.63±0.3	28.75±2.5	45.5±9.47	*P. koraiensis*: *T. amurensis*: *B. costata*: *A. mono*: Other = 3:4:1:1:1
B1	1200	2.13±0.56	24.55±3.3	0.79±0.05	25.8±0.35	28.75±4.79	30.75±9.18	*P. koraiensis*: *L. olgensis*: *P. jezoensis* var. *komarovii*: *A. nephrolepis*: Other = 2:5:1:1:1
B2	1380	1.73±0.18	20.59±1.17	0.79±0.05	21.75±0.65	26.75±4.72	35.5±2.38	*P. jezoensis* var. *komarovii*: *L. olgensis*: *A. nephrolepis*: Other = 4:3:2:1
B3	1560	1.75±0.52	24.42±2.43	0.81±0.04	23.93±0.43	22.5±9.57	26±4.97	*P. jezoensis* var. *komarovii*: *L. olgensis*: *A. nephrolepis*: *B. platyphylla*: Other = 4:2:2:1:1
B4	1670	1.43±0.45	22.63±4.25	0.74±0.06	19.5±0.57	17.75±2.63	27.5±8.66	*P. jezoensis* var. *komarovii*: *L. olgensis*: *A. nephrolepis*: Other = 4:3:2:1
C1	1820	1.4±0.41	22.64±4.15	0.78±0.02	13.3±0.42	8.75±2.5	29.5±1.29	*B. ermanii*: *L. olgensis* = 8: 2
C2	1920	1.03±0.49	22.15±6.43	0.77±0.04	12.63±0.42	6.25±2.5	22.75±5.68	*B. ermanii* 10
	$F_{(8,27)}$	3.47	2.11	2.64	291.70	13.40	5.19	
	p	<0.01	0.0703	<0.05	<0.001	<0.001	<0.001	

Table S2 Transect information of 7 elevations in tundra. The value is the mean and standard deviation of the 4 sample plots at each elevation.

Sample abbreviation	Elevations (m)	Vegetation coverage (%)	Thickness of litter (mm)	Vegetation height (mm)	Vegetation composition (Ratio of area)
D1	2080	100±0	2.00±0.08	9.68±0.33	*Vaccinium uliginosum* Linn.: *Rhododendron confertissimum* Nakai: *Carex pseudo-longerostrata* Chang et Yan = 2:7:1
D2	2180	100±0	2.58±0.21	10.83±0.30	*Vaccinium uliginosum* Linn.: *Rhododendron confertissimum* Nakai : *Dryas octopetala var. asiatica* Nakai: *Carex pseudo-longerostrata* Chang et Yan = 2:6:1:1
D3	2260	98.75±2.50	1.75±0.13	3.95±0.13	*Vaccinium uliginosum* Linn.: *Rhododendron confertissimum* Nakai: *Rhododendron bracteatum* Rehd. et Wils: *Dryas octopetala var. asiatica* Nakai = 3:4:2:1
D4	2340	97.25±1.89	1.78±0.13	2.88±0.30	*Vaccinium uliginosum* Linn.: *Rhododendron confertissimum* Nakai: *Dryas octopetala var. asiatica* Nakai: *Lloydia serotina* (Linn.) Rchb. = 4:4:1:1
D5	2420	96±2.71	1.60±0.08	3.93±0.19	*Rhododendron aureum* Georgi: *Phyllodoce caerulea* (L.) Bab.= 7:3
D6	2530	98±2.16	2.38±0.10	4.53±0.10	*Rhododendron aureum* Georgi: *Rhododendron bracteatum* Rehd. et Wils: *Phyllodoce caerulea* (L.) Bab. = 8:1:1
D7	2600	45±12.91	0.15±0.06	3.53±0.10	*Saussurea tomentosa* Kom.: *Lloydia serotina* (Linn.) Rchb.: *Dryas octopetala var. asiatica* Nakai = 3:5:2
	$F_{(6,21)}$	172.02	811.61	60.67	
	p	<0.001	<0.001	<0.001	

Table S3 Inventory and number of carabid beetles.

Species ID	Species	Individual number
s1	*Agonum bellicum* Lutshnik	2
s2	*Agonum gracilipes* (Duftschmid)	1
s3	*Agonum sculptipes* Bates	2
s4	*Amara erratica* (Duftschmid)	8
s5	*Amara goniodera* Tschitscherine	1
s6	*Amara tumida* Morawitz	7
s7	*Anisodactylus signatus* (Panzer)	1
s8	*Bradycellus glabratus* Reitter	1
s9	*Calosoma cyanescens* Motschulsky	5
s10	*Carabus aurocinctus* Motschulsky	862
s11	*Carabus billbergi* Mannerheim	634
s12	*Carabus canaliculatus* Adams	701
s13	*Carabus constricticollis* Kraatz	20
s14	*Carabus fraterculus* Reitter	82
s15	*Carabus granulatus* Linnaeus	26
s16	*Carabus macleayi* Dejean	249
s17	*Carabus schrencki* Motschulsky	8
s18	*Carabus seishinensis* Lapouge	196
s19	*Carabus venustus* Morawitz	3171
s20	*Carabus vietinghoffi* Adams	46
s21	*Carabus wulffiusi* Morawitz	183
s22	*Chlaenius variicornis* Morawitz	5
s23	*Cychrus morawitzi koltzei* Roeschke	58
s24	*Cymindis vaporariorum* (Linnaeus)	17
s25	*Elaphrus sibiricus* Motschulsky	1
s26	*Harpalus laevipes* Zetterstedt	4
s27	*Harpalus ussuricus* Mlynar	1
s28	*Harpalus ussuriensis* Chaudor	2
s29	*Lachnocrepis prolixa* (Bates)	1
s30	*Leistus janae* Farkac et Plutenko	43
s31	*Leistus niger* Gebler	523
s32	*Loricera pilicornis* (Fabricius)	11
s33	*Miscodera arctica* Paykull	39
s34	*Morphodactyla coreica* (Jedlicka)	587
s35	*Peiyuia* sp.	99
s36	*Nebria ochotica* Sahlberg	5
s37	*Nebria pektusanica* Horratovich	365
s38	*Notiophilus aquaticus* (Linnaeus)	27
s39	*Panagaeus japonicus* Chaudoir	1
s40	*Poecilus lamproderus* (Chaudoir)	13
s41	*Pristosia latistoma* Sasakawa	4
s42	*Pristosia proxima* Morawitz	138
s43	*Pristosia vigil* Tschistcherine	37
s44	*Pterostichus adstrictus* Eschscholtz	1166
s45	*Pterostichus aereipennis* (Solsky)	717
s46	*Pterostichus bellatrix* (Tschitscherine)	8
s47	*Pterostichus comorus* Jedlicka	4103
s48	*Pterostichus eobius* (Tschitscherine)	20

Species ID	Species	Individual number
s49	*Pterostichus gibbicollis* (Mostschulsky)	17
s50	*Pterostichus horvatovichi* Kirschenhofer	197
s51	*Pterostichus interruptus* (Dejean)	433
s52	*Pterostichus jaechi* Kirschenhofer	1055
s53	*Pterostichus jankowskyii* (Tschitscherine)	13
s54	*Pterostichus microps* Heyden	91
s55	*Pterostichus nigrita* (Paykull)	22
s56	*Pterostichus pertinax* (Tschitscherine)	1789
s57	*Pterostichus tuberculiger* (Tschitscherine)	26
s58	*Synuchus agonus* Tschistcherine	6
s59	*Synuchus intermedius* Lindroth	2
s60	*Synuchus melantho* (Bates)	6
s61	*Trichotichnus coruscus* (Tschitscherine)	1
s62	*Xestagonum elytroplanum* Morvan	160
Total		18019
